# Time and cost of linking administrative datasets for outcomes assessment in a follow-up study of participants from two randomised trials

**DOI:** 10.1186/s12874-025-02458-9

**Published:** 2025-01-27

**Authors:** Mohammad Shahbaz, Jane E. Harding, Barry Milne, Anthony Walters, Lisa Underwood, Martin von Randow, Lena Jacob, Greg D. Gamble, for the ANCHOR Study Group, Jane E. Harding, Greg D. Gamble, Caroline A Crowther, Stuart R Dalziel, Carl L Eagleton, Chris JD McKinlay

**Affiliations:** 1 Liggins Institute, The University of Auckland, Auckland, New Zealand; 2 Centre of Methods and Policy Application in Social Sciences, University of Auckland, Auckland, New Zealand

**Keywords:** Record linkage, Feasibility studies, Registries, Costs and cost analysis, Survey and questionnaires, Data management, Information dissemination

## Abstract

**Background:**

For the follow-up of participants in randomised trials, data linkage is thought a more cost-efficient method for assessing outcomes. However, researchers often encounter technical and budgetary challenges. Data requests often require a significant amount of information from researchers, and can take several years to process. This study aimed to determine the feasibility, direct costs and the total time required to access administrative datasets for assessment of outcomes in a follow-up study of two randomised trials.

**Methods:**

We applied to access administrative datasets from New Zealand government agencies. All actions of study team members, along with their corresponding dates, were recorded prospectively for accessing data from each agency. Team members estimated the average time they spent on each action, and invoices from agencies were recorded. Additionally, we compared the estimated costs and time required for data linkage with those for obtaining self-reported questionnaires and conducting in-person assessments.

**Results:**

Eight agencies were approached to supply data, of which seven gave approval. The time from first enquiry to receiving an initial dataset ranged from 96 to 854 days. For 859 participants, the estimated time required to obtain outcome data from agencies was 1,530 min; to obtain completed self-reported questionnaires was 11,025 min; and to complete in-person assessments was 77,310 min. The estimated total costs were 20,827 NZD for data linkage, 11,735 NZD for self-reported questionnaires, and 116,085 NZD for in-person assessments. Using this data, we estimate that for a cohort of 100 participants, the costs would be similar for data linkage and in-person assessments. For a cohort of 5,000 participants, we estimate that costs would be similar for data linkage and questionnaires, but ten-fold higher for in-person assessments.

**Conclusions:**

Obtaining administrative datasets demands a substantial amount of time and effort. However, data linkage is a feasible method for outcome ascertainment in follow-up studies in New Zealand. For large cohorts, data linkage is likely to be less costly, whereas for small cohorts, in-person assessment has similar costs but is likely to be faster and allows direct assessment of outcomes.

## Background

For follow-up studies of participants in randomised trials, data linkage appears to be a plausible and less costly method for obtaining a wide range of outcome measures [1]. Linked administrative data can enhance the usage of existing data, avoid duplicate primary data collection, and reduce the cost of data acquisition. Data linkage to administrative datasets may also overcome challenges faced by traditional methods of collecting outcome data, such as the cost of tests, the need for human resources for data collection, missing data (although data may also be missing from administrative datasets) and difficulties in participants providing accurate and complete data [2]. 

While these benefits of linking administrative datasets appear attractive, researchers intending to use this approach often encounter technical and budgetary challenges [3]. Data requests often require a significant amount of information from researchers and are frequently duplicated across various application formats [4, 5]. The process for data acquisition from several national datasets to follow up study participants in the UK took several years, requiring numerous meetings, emails, and telephone calls [4, 6]. The high costs associated with data access, coupled with the complexities of navigating data access agreements and procedures, may also hinder studies seeking to use administrative datasets [7, 8]. While randomised pragmatic trials that only require a single administrative dataset have reported enhanced trial outcomes assessment using this method [9], those requiring a broader range of outcomes must link records from multiple datasets, potentially resulting in significant costs and time investment [10].

Individuals in New Zealand are allocated a person-specific identifier for health service interactions (the National Health Index, NHI), and there are established government data collection and coding protocols. This makes linkage to administrative datasets an apparently attractive option for determining outcomes in adulthood after a perinatal randomised trial. However, the processes and resource implications for doing this have not been described previously. Therefore, this study aimed to describe the feasibility, direct costs and total time required to obtain datasets for the assessment of outcomes (cardiometabolic risk, respiratory, general health, mental health, educational and social outcomes) in a follow-up study of adult health and wellbeing after two randomised trials of exposure to antenatal corticosteroids [11, 12]. Additionally, we compared the costs and time associated with linkage to administrative datasets with those estimated for obtaining similar outcomes using self-reported questionnaires and conducting in-person assessments.

## Methods

The ANCHOR study was a follow-up study of two randomised trials investigating the long-term effects of antenatal corticosteroids. In the Auckland Steroid Trial [13] consenting women expected to deliver between 24 and 36 weeks’ gestation between December 1969 and February 1974 at National Women's Hospital, Auckland, New Zealand were randomly assigned to receive intramuscular betamethasone or placebo. The trial included 1,115 women and 1,218 infants [11]. In the ACTORDS study [14] consenting women who remained at risk of preterm birth at less than 32 weeks’ gestation, seven or more days after receiving a first course of antenatal corticosteroid, between April 1998 and July 2004 at 16 hospitals in Australia and seven in New Zealand, were randomly assigned to receive an intramuscular dose of either betamethasone or placebo, repeated every week until delivery. The ACTORDS trial included 982 women and 1,146 babies.

All surviving children and grandchildren of participants in the Auckland Steroid Trial, and all surviving children of participants in the ACTORDS Trial who were randomised in New Zealand were eligible to participate in this follow-up study. Potential participants were traced, asked to complete a questionnaire, and also asked for consent for the research team to obtain their data from various agencies. Participants from the Auckland Steroid Trial were recruited for the follow-up study between March 3, 2021, and May 31, 2022, while participants from the ACTORDS study were recruited between January 13 and October 19, 2022. The outcomes of interest included a broad range of health measures including cardiometabolic risk, respiratory conditions, and mental health, as well as social and economic measures.

The agencies from which data were sought included the New Zealand Ministries of Health, Education, Justice, Disabled People, and Social Development, HealthAlliance TestSafe data (laboratory test results from community laboratories in the Northern Region Health District of New Zealand), the Accident Compensation Corporation, the B4 School Check (health and development data for 4 to 5 year-olds from the Ministry of Health), and the New Zealand Qualifications Authority. Participants could choose which, if any, dataset(s) they consented to for record linkage. The study team applied to each agency for data access (Table 1). For successful applications, lists of participant identifiers for consenting participants were provided to the agencies, including NHIs for health data, National Student Numbers (NSNs) for education and qualification data, and name, sex, date of birth, and address for probabilistic matching of justice and education data where NSNs were not available (Table 1).

**Table 1:** Procedure for data access

Data Sources	Initial contact	Application process	Approval process	Data format	Matching identifier
*Ministry of Health (national health data)	One of the team members had a previous contact with the agency or had applied for data before	Letter, copy of protocol and ethics approval	In house data review committee. The agreement and cost estimate was produced by the agency	Comma delimited text files with password protection	***NHI
Ministry of Health (B4 school data)	One of the team members had a previous contact with the agency or had applied for data before	Application forms sent by the agency	In house data review committee	Comma delimited text files with password protection	NHI
Accident Compensation Corporation (accident and injury data)	Contact information was obtained from the agency's website	Online submission form and ethics application form	In house data review and ethics committee. List of required variables sent to the agency for approval. Minor modification of agreement suggested by the agency	Comma delimited text files with password protection	NHI
** Ministry of Disabled Peoples (Whaikaha) (disability data)	Contact information was obtained from the agency's website	Copy of participant information sheet, consent documents and protocol	In house data review committee and ethics advisor	Comma delimited text files	NHI
HealthAlliance TestSafe (laboratory data)	One of the team members had a previous contact with the agency or had applied for data before. The email contact information was obtained from the agency's website	Letter, copy of protocol and ethics approval	Data request discussed in internal monthly meeting and in house committee approved application. After the approval a meeting was scheduled between one of the research team members and the manager to discuss the timeline and project requests	Comma delimited text files with password protection (aggregated electronic records plus non-coded free text descriptions of numeric variables)	NHI
Department of Internal Affairs (death certificates)	Contact information was obtained from the agency's website	Online submission form/ Email contact	In house data review	Comma delimited text files with password protection	NHI if available or, name, sex, date of birth
Ministry of Education (support and stand down data)	One of the team members had a previous contact with the agency	Letter, copy of protocol and ethics approval	Memorandum of Understanding drafted by the agency and reviewed and revised by university legal team. In house ethics reviewer	Comma delimited text files with password protection	****NSN and names, sex, date of birth
Ministry of Education (New Zealand Qualifications Authority)	Contact information was obtained from the agency's website	Letter, copy of protocol and ethics approval	Memorandum of Understanding drafted by the agency and reviewed and revised by university legal team	Comma delimited text files with password protection	NSN if available or probabilistic matching of name, sex, date of birth, previous schools
Ministry of Justice (convictions data)	Contact information was obtained from the agency's website	Application forms sent by the agency	Memorandum of Understanding drafted by the agency and reviewed and revised by university legal team	Individual PDF transcript of each participant record attached to separate emails	Probabilistic matching of name, sex, date of birth and address where available
Ministry of Social Development (Social benefits data)	Contact information was obtained from the agency's website	Letter, copy of protocol and ethics approval	Agency aimed to draft Memorandum of Understanding. Privacy Principles details provided by research team	Data request declined	-

^*^Ministry of health provided the cancer registry, the virtual diabetes register, hospital admission (National Minimum dataset), clinic attendance (National Non-Admitted Patient Collection), and pharmaceutical data

^**^Data were held previously by the Ministry of Health

^***^NHI, National Health Index number (a unique health identifier for anyone who has had any contact with the New Zealand health system)

^****^NSN, national student number

All actions of study team members, along with their corresponding dates, were recorded prospectively for each agency. These included initial enquiries, follow-up emails, phone calls, meetings, sending requested documents, replies from agencies, and data receipt. The team met weekly to discuss progress with dataset requests, and to capture dates and actions.

To estimate the time required for each action related to data linkage, team members provided the average time they spent on each action. We made the conservative assumptions that, on average, sending a follow-up email for a requested file (not including preparation time) required five minutes; a phone call, 15 min; an online video meeting, 30 min; an in-person visit, 120 min; and review of an application form or contract, 60 min.

Fees charged for providing the requested data were derived from the invoices paid to each agency. We have not yet received invoices for Ministry of Health B4 School Check data, Accident Compensation Corporation data, Ministry of Disabled People disability data, Ministry of Education support and stand-down data, or New Zealand Qualifications Authority data, but assume that the fees would be comparable to the fee charged by the Ministry of Health for national health datasets, and that this would not differ with cohort size. The Ministry of Justice and Department of Internal Affairs charged on a per participant basis.

Similarly, all actions of study team members and the dates relating to obtaining questionnaires were recorded prospectively, including contacts with participants such as e-mails, text messages, phone calls and in person visits if other methods of contact were not successful. We made conservative assumptions that, on average, sending an email, letter, or text required five minutes, a phone call 30 min, and a home visit (used when other methods of contact failed) necessitated 150 min, based on recall audit of average times reported by study team members.

For in-person assessments, we assumed that the time needed for arranging and conducting the assessments and completing a nurse-assisted questionnaire would be 90 min, based on our previous experience of similar assessments. We used the published costs of laboratory tests in Auckland [15–17].

To estimate the costs of staff time, we multiplied the time required for actions by the mid-point wage of a senior registered nurse [18], which was 64 New Zealand dollars (NZD, equivalent to 39 USD at time of writing) per hour. To calculate the total costs for data linkage, we included both fees charged by agencies and staff costs. For self-reported questionnaires, we calculated staff costs only. For in-person assessments, we included the costs of laboratory tests and staff costs. For comparison with other methods, we calculated the number of actions and the required time and cost for those actions for all consenting participants.

### Non-documented actions

The actions required to obtain the study outcomes data were not all documented prospectively and therefore could not be included in this analysis. The time required to obtain consent from potential participants was excluded from all calculations because consent was a prerequisite for all outcome ascertainment methods. In addition, for data linkage, the non-documented actions included weekly team meetings, legal review of contracts, extracting participant identifiers for consenting participants to send to each agency, coding of medications, assigning ICD-10-AM codes to outcome definitions, converting data formats, coding free text records, categorising laboratory test data labels according to test type and units, and matching the diagnostic codes for participant data with ICD-10-AM codes in outcome definitions.

For self-reported questionnaires, the non-documented actions included the time spent on setting up the questionnaires online, data management, monitoring, and quality control. For in-person assessments, the non-documented actions included transport to the study site, preparing equipment and arranging transfer of samples to the laboratory, data entry, monitoring, quality control, and the costs of consumables.

### Descriptive economic analysis

For data linkage, we calculated the number of contacts, time, and cost required to obtain data from each agency, beginning from the date of the first enquiry. We calculated the time from enquiry to application approval, and then from application approval to receiving the first data, and final data, excluding the periods when we were recruiting participants and the agency was waiting for participant identifiers to be sent.

For the self-reported questionnaire, we calculated the number of contacts, and hence time and cost that the study team spent to obtain completed self-reported questionnaires for all participants. For in-person assessments, we estimated the time and cost required to complete the assessments for all participants. SAS (v9.4 SAS Institute Inc, Cary NC) statistical software was used for data analysis and preparing figures.

### Study outcomes

Time and cost required to obtain participants’ data from:

1. Data linkage (each administrative dataset):

- Time from the initial contact to the contract, and from the contract to the delivery of initial and final datasets
- Number of contacts by study team members
- Direct costs of obtaining dataset
- Estimated total costs of obtaining dataset

2. Self-reported questionnaires

- Number of contacts by study team members and the estimated time for each contact
- Estimated total costs of obtaining completed questionnaires

3. Estimated time and cost of assessing study outcomes.

### Sensitivity analyses

To assess the robustness of the total time and therefore cost estimates to variation in the time taken for various actions, the time required for each action was doubled (worst case scenario) and halved (best case scenario).

To test the sensitivity of estimated time and costs to cohort size, we estimated the total time and costs of data collection for cohorts of 100 participants and 5,000 participants.

## Results

In the follow-up study, we applied to obtain information for 859 participants who consented, including 424 adult offspring (mean age 49.3 years, SD 1.0, 50% female) and 221 grandchildren (mean age 16.5 years, SD 6.0, 54% female) of mothers who took part in the Auckland Steroid Trial, as well as 214 offspring (mean age 20.5 years, SD 1.5, 45% female) of mothers who took part in the ACTORDS trial. Of the 859 who consented, 103 (12%) were living overseas. We obtained administrative data on 829 participants (96%), and a questionnaire was completed by 734 participants (85%).

Of the eight agencies from which data were sought, seven approved the application, and only the Ministry of Social Development declined, citing a lack of capacity to undertake such a major task (Table 2). From 23 data requests we received 30 batches of data for 13 different datasets. Each agency had a different set of processes for completing and reviewing applications, and often opaque points of contact. Covid pandemic, inexperience with research requests and substantial pressures from ongoing health reforms contributed to delays.

**Table 2:** The time and cost required to obtain participants’ data from each administrative data source (*n* = 859)

Data Sources	Time from enquiry to application decision (days)	*Time from application approval to receiving first data (days)	Time from first identifiers sent to receiving first data (days)	*Time from enquiry to receiving first data (days)	*Time from enquiry to receiving final data (days)	Number of follow-up emails	Number of file requests from agencies	**Number of phone calls/texts	Number of meetings	Number of replies from agency	Quoted cost for complete data delivered (NZD)	Time required for all staff actions. (minutes)	#Total costs (NZD)
Ministry of Health (national health data)	288	133	62	421	686	17	2	-	-	17	862	155	1027
Ministry of Health (B4 School Check)	34	62	27	96	142	2	1	-	-	3	***862	75	942
Accident Compensation Corporation data	370	71	4	441	467	13	4	-	2	18	***862	205	1081
Ministry of Disabled Peoples disability data	156	300	173	456	456	19	6	1/2	2	17	***862	330	1214
HealthAlliance TestSafe laboratory data	48	100	35	148	452	12	2	-	3	17	4844	220	5079
Department of Internal Affairs (death certificates)	-	-	33	1	128	5	1	-	-	6	956	90	1052
Ministry of Education (support and stand down data)	452	402	7	854	854	24	7	-	-	26	***862	215	1091
Ministry of Education (New Zealand Qualifications Authority)	105	96	86	201	318	8	3	1/0	-	12	***862	130	1000
Ministry of Justice convictions data	88	26	37	114	213	3	3	-	-	5	8,224	90	8320
Ministry of Social Development social data***	-	-	-	-	-	2	2	-	-	6	-	20	21
Total	-	-	-	-	-	105	31	2/2	7	127	19,196	1530	20,827

^*^Time calculated excluding periods when we were recruiting participants and the agency was waiting for participant identifiers to be sent

^**^Phone calls only included where we were able to talk, excluding unanswered calls

^***^Invoice not yet received; the estimated cost was 862 NZD. #Based on 64 NZD per hour. See methods for excluded costs

^***^Request declined due to lack of capacity

**Fig. 1:**
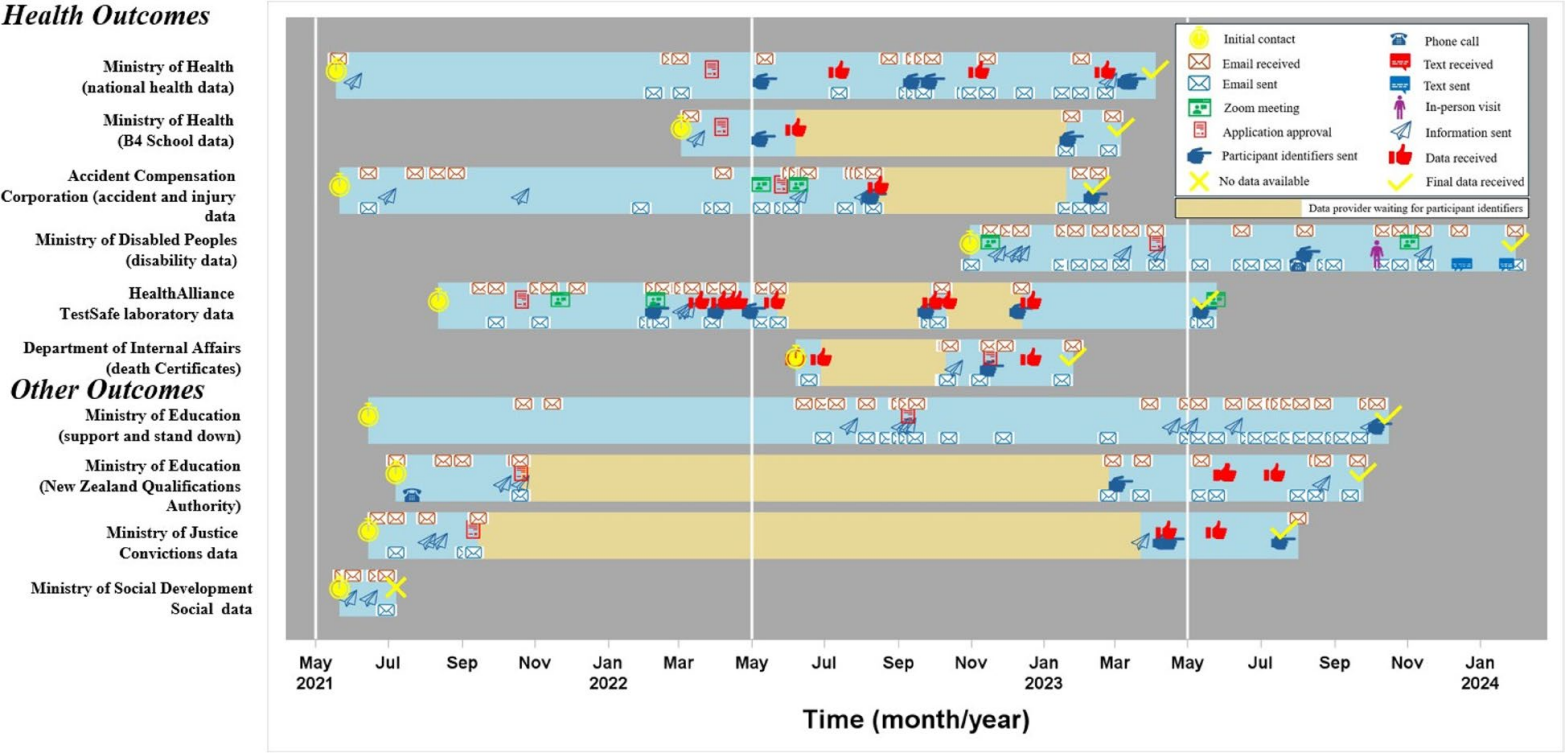
Timeline of the data linkage process, from the initial enquiry to data acquisition

Obtaining all the data from the agencies for this study took two years and four months. All agencies except the Ministry of Education quickly replied after initial enquiry. Over this period when many data acquisition processes were happening simultaneously, the number of follow-up emails sent to any agency ranged from two to 24 and the number of replies from agencies ranged from three to 26. The number of file requests made by agencies ranged from one to seven.” The time from first enquiry to application approval ranged from 34 days for Ministry of Health B4 School Check data to 452 days for Ministry of Education support and stand down data, with a mean of 193 (SD 158) days across the eight agencies. (Table 2, Fig. 1). The process of data acquisition, from the first enquiry to receiving the first datasets, ranged from 96 days for the Ministry of Health B4 School Check data to 854 days for the Ministry of Education's support and stand-down data (mean (SD) 304 (266) days). The time from first matching identifier sent to receiving an initial dataset ranged from 4 days for Accident Compensation Corporation data to 173 days for Ministry of Disabled People disability data (mean 52 (52) days). The total time required to obtain participants’ data from the initial enquiry to receiving the final data ranged from 128 days for death certificates to 854 days for Ministry of Education support and stand down data (mean 413 (245) days). Over this period, the number of follow-up emails sent to any agency ranged from two to 24 and the number of replies from agencies ranged from three to 26. Because not all agencies were able to match data on the basis of the national health identifier, between one and five separate participant identifiers were supplied to the various agencies for record linkage. The time required for all actions to obtain participant data from various agencies ranged from 75 min for Ministry of Health B4 School Check data to 220 min for HealthAlliance TestSafe laboratory data (Table 2, Fig. 1).

We made multiple data requests to some agencies, and receiving multiple data extracts was helpful for discovering any data integrity issues. For instance, we received multiple data extracts from HealthAlliance Testsafe, and from the last extract, we discovered that a data extraction algorithm was selectively excluding some records. We had accessed the clinical records and randomly checked a subset of participants who had records in the TestSafe laboratory dataset to see if they had additional earlier or later records, and found that some had more records in TestSafe than we had been sent. After investigation, we found that this occurred because these individuals had multiple national health index numbers. We had provided the TestSafe with a single national health index number, and TestSafe provided records associated only with that specific number rather than all data associated with the participant. Upon enquiry, TestSafe discovered that a filter excluding secondary national health index numbers had been incorrectly applied, which led to the missing records. After resolving the issue, we requested a complete re-extraction of all TestSafe data.

The total time required for all actions needed for data linkage for 859 participants was 1,530 min (Table 2). In contrast, the estimated time required to obtain completed self-reported questionnaires for this number was 11,025 min (Table 3) and to complete in-person assessments was 77,310 min (Table 4). This was the equivalent of less than two minutes per participant for data linkage, 13 min per participant for obtaining completed self-reported questionnaires, and 90 min per participant for in-person assessments (Tables 2 , 3 and 4). The estimated total cost of linking datasets was 20,827 New Zealand Dollars (NZD), for obtaining completed self-reported questionnaires was 11,735 NZD, and for in-person assessments was 116,085 NZD (Tables 2, 3 and 4).

**Table 3:** The time and estimated costs required to obtain completed self-reported questionnaires from all participants (*n* = 859)

	Total number of contacts	Total time (minutes)	Time per participant (minutes)	*Total costs (NZD)
Email	1,019	5,095	6	5,431
Letter	29	145	0	155
Mobile text	139	695	1	741
Phone call	112	3,360	4	3,582
Voicemail (phone call)	11	330	0	352
Facebook direct	9	45	0	48
Instagram direct	0	0	0	0
Home visit	9	1,350	2	1,439
LinkedIn direct	0	0	0	0
Zoom	0	0	0	0
Other	1	5	0	5
Total	1,331	11,025	13	11,753

Estimated time required for an email, letter, or text 5 min, a phone calls 30 min, and a home visit 150 min.* Based on 64 NZD per hour. See methods for excluded costs

**Table 4:** The estimated time and estimated cost required for in-person assessment of all participants (*n* = 859)

	Each participant	Total (*n* = 859)
Time (minutes)	90	77,310**
*Haemoglobin A1c (HbA1c)	22.45	19,285
*Glucose	3.28	2,818
*Lipids – Fasting	13.41	11,519
*Total cost of Laboratory tests	39.14	33,621
Total costs	135.14	116,085**

^*^The cost is in NZD

^**^ Based on 64 NZD per hour. See methods for excluded costs

### Sensitivity analyses

In sensitivity analyses, when the estimated time for each action was doubled, the total time to obtain participant data from various agencies was 3,060 min, to obtain completed self-reported questionnaires was 22,050 min, and for in-person assessments was 154,620 min (Table 5 and 6). The total cost to obtain participant data from agencies was 22,458 NZD, to obtain completed self-reported questionnaire was 23,505 NZD, and for in-person assessments was 198,446 NZD.

**Table 5:** The time and cost required to obtain participants’ data from administrative data sources (sensitivity analyses)

Data Sources	Time required for all actions halved (minutes)	Time required for all actions doubled (minutes)	Total cost (NZD) for 100 participants	Total cost (NZD) for 5000 participants
Ministry of Health (national health data)	78	310	862	862
Ministry of Health (B4 school data)	38	150	*862	*862
Accident Compensation Corporation data	103	410	*862	*862
Ministry for Disabled Peoples disability data	165	660	*862	*862
HealthAlliance TestSafe laboratory data	110	440	4,844	4,844
Department of Internal Affairs (death certificates)	45	180	111	5,564
Ministry of Education (support and stand down data)	108	430	*862	*862
Ministry of Education (New Zealand Qualifications Authority)	65	260	*862	*862
Ministry of Justice convictions data	45	180	1,100	55,000
Ministry of Social Development social data	10	40	-	-
Total	765	3,060	11,227	70,580

^*^Invoice not yet received; the estimated cost was 862 NZD. See methods for excluded costs

**Table 6:** The time and estimated costs required for different outcome ascertainment methods and sensitivity analyses

	Actual costs (NZD)	Staff time (minutes)	Estimated costs of staff time (NZD)	Total costs (NZD)
**This Study (*n* = 859)** Data linkage	19,196	1,530	1,631	20,827
Self-reported questionnaire	-	11,025	11,753	11,753
In-person assessments	33,621	77,310	82,464	116,085
**Sensitivity analyses, time doubled** Data linkage	19,196	3,060	3,262	22,458
Self-reported questionnaire	-	22,050	23,505	23,505
In-person assessments	33,621	154,620	164,825	198,446
**Sensitivity analyses, time halved** Data linkage	19,196	765	815.5	20,011.5
Self-reported questionnaire	-	5,512.5	5,876.5	5,876.5
In-person assessments	33,621	38,655	41,232	74,853
**Sensitivity analyses for 100 participants** Data linkage	11,227	1,530	1,631	12,858
Self-reported questionnaire	-	1,283	1,368	1,368
In-person assessments	3,914	9,000	9,594	13,508
**Sensitivity analyses for 5000 participants** Data linkage	70,580	1,530	1,631	72,211
Self-reported questionnaire	-	64,173	68,408	68,408
In-person assessments	195,700	450,000	479,700	675,400

^*^Based on 64 NZD per hour. See methods for excluded costs

When the estimated time for each action was halved, the total time to obtain participant data from various agencies was 765 min, to obtain completed self-reported questionnaires was 5,513 min, and for in-person assessments was 38,655 min (Table 6). The total cost to obtain participant data from agencies was 20,012 NZD, to obtain completed self-reported questionnaire was 5,877 NZD, and for in-person assessments was 74,853 NZD.

The estimated total time required for obtaining data for a cohort of 100 participants was 1,530 min through data linkage, 1,283 min through self-reported questionnaires, and 9,000 min through in-person assessments. The estimated total cost was 12,858 NZD for data linkage, 1,368 NZD for obtaining completed self-reported questionnaires, and 13,508 NZD for in-person assessments (Table 6).

The estimated total time required for obtaining data for a cohort of 5000 participants was 1,530 min through data linkage, 64,173 min through self-reported questionnaires, and 450,000 min through in-person assessments. The estimated total cost was 72,211 NZD for data linkage, 68,408 NZD for self-reported questionnaires, and 675,400 NZD for in-person assessments (Table 6).

## Discussion

We aimed to determine the total time and costs of accessing administrative datasets for assessment of chronic condition outcomes in a follow-up study of adult health and wellbeing after two randomised trials of exposure to antenatal corticosteroids. We found that applying for datasets from agencies was not a straightforward process, demanding a substantial amount of time and involving a considerable number of actions. There were many differences in the requirements of the various agencies and in their experience in dealing with data sharing requests.

Delay in accessing datasets challenges timely research and policymaking [5]. Similar to our findings, a UK study reported that the process of obtaining administrative data for linking five national datasets was exceedingly intricate, labour-intensive, and occasionally disheartening, and lead to delays in scientific progress [4]. Another study conducted in England which successfully linked health and education data highlighted that the evolving regulations on personal data usage and the absence of precedents demanded significant time, careful effort, and expertise from both researchers and data providers to access administrative data for research purposes [6]. Data record linkage in Australia was also reported to be complex, and to require substantial time and financial cost [19]. In contrast to the situation in United Kingdom and Australia, New Zealanders have a unique national health identifier which facilitates record linkage across routinely collected health datasets. National records exist for hospital admissions, mortality and pharmaceuticals. There is a commitment to data sharing across administrative platforms where informed participant consent is provided. Despite these advantages the process of data linkage is complex and quite lengthy.

Our study showed that, although data linkage for 859 participants across various agencies took two years and four months, it was a feasible method for outcome ascertainment in the follow-up of randomised trials in New Zealand. The agencies that approved our request successfully provided participants’ data. We obtained data on almost all participants who consented to participate, including some who lived outside New Zealand, and this was usable for our research purposes.

Data linkage required less staff time than other methods, and offers a broad range of outcomes, depending on the existence of participant identifiers, and completeness, accuracy, consistency, and compatibility of data sources. In addition, it has inherent advantages, such as reducing recall and ascertainment bias, providing comprehensive data on hard-to-reach sub-populations, and minimal burden on participants [20, 21]. Studies requiring the linkage of fewer data sources, such as those focused solely on clinical outcomes, would also likely be able to acquire their data more rapidly than we did. In particular, we found the acquisition time for health datasets was nearly half that of other datasets, and if only health datasets were needed, the cost of data linkage could be reduced by half.

Administrative data obtained for our study may have been incomplete or contained errors. For example, TestSafe laboratory data were incomplete when first received; a problem that was only identified and resolved after spot-checks against individual participant records, considerable exploration of the data and meetings with the TestSafe team. Using the complete dataset, we identified two new cases of diabetes, and 38 additional cases of hyperlipidaemia. This required us to re-adjudicate the outcomes and re-run the statistical analyses, although the findings of the follow-up study remained unchanged. While this issue did not affect the findings in this instance, in other cases it could potentially alter the results, especially for low prevalence outcomes such as diabetes.

We obtained completed self-reported questionnaires from 85% of our participants. Although, for comparison with other methods, we estimated and reported the time and cost required to obtain questionnaires for all participants, in practice the rate of obtaining completed self-reported questionnaires may vary and is very unlikely to be 100%, thus potentially reducing the time and cost involved. However, missing data in outcome assessments may introduce bias, reduce statistical power, and affect the generalisability of findings. In addition, while obtaining self-reported questionnaires may be a cheaper and quicker method, especially for smaller cohorts, our previous findings indicated that agreement between self-reported questionnaires and data linkage varied for chronic conditions [12], potentially resulting in an underestimation of the prevalence of health conditions among follow-up cohorts when using self-reported questionnaires alone. While administrative datasets also underestimated the prevalence of chronic conditions, administrative data alone identified 2–3 times more cases of diabetes, pre-diabetes, hyperlipidemia, and asthma compared to questionnaires, except for hypertension and mental health disorders, where the questionnaire identified 1–2 times more cases than administrative data. Combining self-reported questionnaires with data linkage appears to improve outcome ascertainment and increase study power [12].

Our data suggest that for large cohorts in-person assessments are likely to be much more expensive than data linkage, potentially rendering this method less feasible in some cases. In addition, some participants may be living overseas, and not all are likely to be willing or able to attend an in-person assessment, potentially resulting in assessment of study outcomes for a smaller proportion of the total cohort, although this may also apply to data linkage. However, for small cohorts, we estimated that the cost of in-person assessments was comparable to that of data linkage. Further, with appropriate staffing, these assessments could be completed much faster than the prolonged process of data linkage. In-person assessment also allows direct measurement of the outcomes of interest, eliminating the need to extrapolate the absence of data in linked datasets as indicative of the absence of the outcome.

The costs presented in this study underestimate the costs required for all methods, as we estimated the average time spent for each action and excluded many activities necessary for obtaining usable datasets. For example, for self-reported questionnaires, we only included staff costs and omitted expenses related to setting up the questionnaires online, data management, and data monitoring, which may incur significant costs. Additionally, preparing linked datasets for analysis requires substantial time and thus costs.

We also excluded the time and cost required to obtain consent from potential participants, as it is a prerequisite for all three methods. In our study, the staff time required for this phase was three and a half times longer than for obtaining completed self-reported questionnaires (an average of 45 min per participant), and therefore needs to be considered in planning timelines for future studies.

Future studies may also benefit from understanding the types of documents agencies require and that different agencies have distinct application processes. Although this study is specific to the New Zealand setting, many countries have nationwide repositories of administrative datasets, and the process of applying to agencies, including initial inquiry, application submission, in-house review, ethics review, approval, and contracting, is likely to be similar across countries. Additionally, the relative time and cost involved in obtaining self-reported questionnaires and conducting in-person assessments is likely to be comparable internationally.

### Strengths of the study

All steps in the processes of accessing datasets and obtaining questionnaires were documented prospectively. In addition, a senior researcher was involved in contacting all agencies. This team member had previous contact with some of the agencies, was familiar with data request processes, and mainly worked with national datasets for data linkage studies. This experience helped us find the right contact person in each agency more easily. We assume that without an experienced senior research member, considerable staff training would have been required and it could have taken even longer to access the datasets. The team met weekly to review progress and prompt additional actions or alternative approaches if needed. In addition, we had one clinician involved in reviewing the data extracts received from agencies to ensure completeness and readiness for analysis. This review was particularly beneficial in identifying and addressing small errors in the data extracts.

### Limitations of the study

We did not document all actions and actual time required for them prospectively, because we had not planned a complete economic analysis at the time of study design. Therefore we were not able to include those actions in the analysis, and this is likely to have resulted in a substantial underestimate of total costs and time required, particularly for data linkage.

Some participants may be living overseas, and not all are likely to be willing or able to attend an in-person assessment, potentially resulting in assessment of study outcomes for a smaller proportion of the total cohort. Similarly, those participants living overseas may not have complete data in administrative datasets or may have no data at all.

These results reflect contacts made during a time of health restructuring in New Zealand and represent a snapshot in time. Our prior experience with data linkage requests from the Ministry of Health has been that the time to provide data varies widely, apparently depending on workload and staffing in the Ministry. Except for the late December through early January summer holiday period we found no evidence that calendar month had an impact on any contact timings. Whaikaha, the Ministry for Disabled Peoples was newly established. Thus, the contact times reported here may not necessarily be representative of future enquiries.

## Conclusion

Applying for administrative datasets from New Zealand agencies is not a straightforward process; it demands a substantial amount of time and effort. However, data linkage is a feasible method for outcome ascertainment in New Zealand follow-up studies. For large cohorts, it is likely to be less costly compared to in-person assessments. In-person assessments are likely to be preferred for small cohorts as they have similar costs but are able be completed faster and allow for direct assessment of outcomes of interest.

## Data Availability

De-identified participant data will be available to researchers who provide a methodologically sound proposal with appropriate ethical approval, where necessary, and following approval of the proposal by the Data Access Committee of the Liggins Institute. Data requestors will be required to sign a data access agreement before data are released. Request for access to data can be made to the Maternal and Perinatal Research Hub at the Liggins Institute, University of Auckland (researchhub@auckland.ac.nz).

## Abbreviations

NHI: National Health Index
ACTORDS: Australasian collaborative trial of repeat doses of steroids
NSN: National Student Number
NZD: New Zealand Dollar
